# Alterations of gut microbiota are associated with blood pressure: a cross-sectional clinical trial in Northwestern China

**DOI:** 10.1186/s12967-023-04176-6

**Published:** 2023-06-30

**Authors:** Jing Lv, Jihan Wang, Yan Yu, Mengyao Zhao, Wenjuan Yang, Junye Liu, Yan Zhao, Yanjie Yang, Guodong Wang, Lei Guo, Heping Zhao

**Affiliations:** 1 Department of Clinical Laboratory, Honghui Hospital, Xi’an Jiaotong University, Xi’an, China; 2 Institute of Medical Research, Northwestern Polytechnical University, Xi’an, China; 3 Department of Cardiology, The First Affiliated Hospital of Xi’an Jiaotong University, Xi’an, China; 4 Department of Quality Control, Xi’an Mental Health Center, Xi’an, China; 5 Department of Spine Surgery, Honghui Hospital, Xi’an Jiaotong University, Xi’an, China

**Keywords:** Blood pressure, Gut microbiota, Hypertension, Metagenome, Northwestern China, Sex difference

## Abstract

**Background:**

The human gut microbiota (GM) is involved in the pathogenesis of hypertension (HTN), and could be affected by various factors, including sex and geography. However, available data directly linking GM to HTN based on sex differences are limited.

**Methods:**

This study investigated the GM characteristics in HTN subjects in Northwestern China, and evaluate the associations of GM with blood pressure levels based on sex differences. A total of 87 HTN subjects and 45 controls were recruited with demographic and clinical characteristics documented. Fecal samples were collected for 16S rRNA gene sequencing and metagenomic sequencing.

**Results:**

GM diversity was observed higher in females compared to males, and principal coordinate analysis showed an obvious segregation of females and males. Four predominant phyla of fecal GM included Firmicutes, Bacteroidetes, Actinobacteria and Proteobacteria. LEfSe analysis indicated that phylum unidentified_Bacteria was enriched in HTN females, while Leuconostocaceae, *Weissella* and *Weissella_cibaria* were enriched in control females (P < 0.05). Functionally, ROC analysis revealed that Cellular Processes (0.796, 95% CI 0.620 ~ 0.916), Human Diseases (0.773, 95% CI 0.595 ~ 0.900), Signal transduction (0.806, 95% CI 0.631 ~ 0.922) and Two-component system (0.806, 95% CI 0.631 ~ 0.922) could differentiate HTN females as effective functional classifiers, which were also positively correlated with systolic blood pressure levels.

**Conclusions:**

This work provides evidence of fecal GM characteristics in HTN females and males in a northwestern Chinese population, further supporting the notion that GM dysbiosis may participate in the pathogenesis of HTN, and the role of sex differences should be considered.

*Trial registration *Chinese Clinical Trial Registry, ChiCTR1800019191. Registered 30 October 2018 – Retrospectively registered, http://www.chictr.org.cn/.

**Supplementary Information:**

The online version contains supplementary material available at 10.1186/s12967-023-04176-6.

## Background

As a common and chronic medical condition, hypertension (HTN) has become a global health issue, accounting for approximately 10.8 million deaths worldwide [1]. HTN is a complex and modifiable risk factor for cardiovascular diseases (CVDs) and stroke, while a diverse range of endogenous and environmental factors contributes to both HTN onset and progression [2–4]. Although an increased risk of HTN has been demonstrated to be associated with approximately 900 genetic loci, only < 6% of the variance in systolic blood pressure (BP) could be explained by common genetic risk variants [5]. Given its complexity and heterogeneity, the elucidation of HTN pathogenesis remains challenging.

Growing evidence has revealed the potential role of the gut microbiota (GM) in host homeostasis and multiple physiological processes [6], and suggests associations between GM and various diseases including atherosclerotic CVDs [7]. As the longest organ, the gastrointestinal tract is mainly involved in the absorption of nutrients and ions which greatly impacts BP [8], and a substantial amount of work has supported the role of GM as a potential factor in BP regulation or even causal determinants of HTN pathogenesis [2, 9–11]. There is evidence of gastrointestinal pathophysiology in animal HTN models, in which fecal transplantation from HTN subjects increases BP in germ-free mice [12]. In addition, GM dysregulation is also associated with various metabolic diseases and HTN-related risk factors, such as obesity, hyperlipidemia and diabetes mellitus [13]. Overall, current data strongly indicate that GM may play an important role in HTN pathogenesis [12, 14–16].

Due to the novel concept of GM-influenced HTN pathogenesis [2], it is critical to investigate GM alterations in HTN subjects in different regions with different genetic background and dietary habits [2, 16–19]. Moreover, obesity, hyperlipidemia and diabetes could result in GM alterations [20], and these variations should also be taken into consideration. Of note, few studies have investigated the associations between GM and HTN subjects based on sex differences. Given that sex is important in BP regulation [2, 21, 22], we investigated the GM characteristics of female and male HTN subjects, respectively, and the associations between GM characteristics and BP levels were discussed.

## Methods

### Study participants and study protocol

From July 2018 to June 2020, we recruited 205 subjects from our outpatient clinics at Honghui Hospital, Xi’an Jiaotong University, China. All selected subjects were aged between 18 and 80 years old, and were able to provide written informed consent. Subjects with one of the following conditions were excluded: ① pregnant or lactating, or with chronic diseases including cancers, inflammation or surgical history in the alimentary tract, or serious systematic dysfunctions; ② taking any medications that may disrupt their original GM, such as fiber supplements, probiotics or prebiotics within 6 weeks, or antimicrobial drugs within 6 months before GM sampling [2, 23]; ③ taking any anti-inflammatory agents, acid-suppressing agents, immunosuppressants, or anti-HTN medication, which seem to modulate GM [2, 24, 25].

**Fig. 1:**
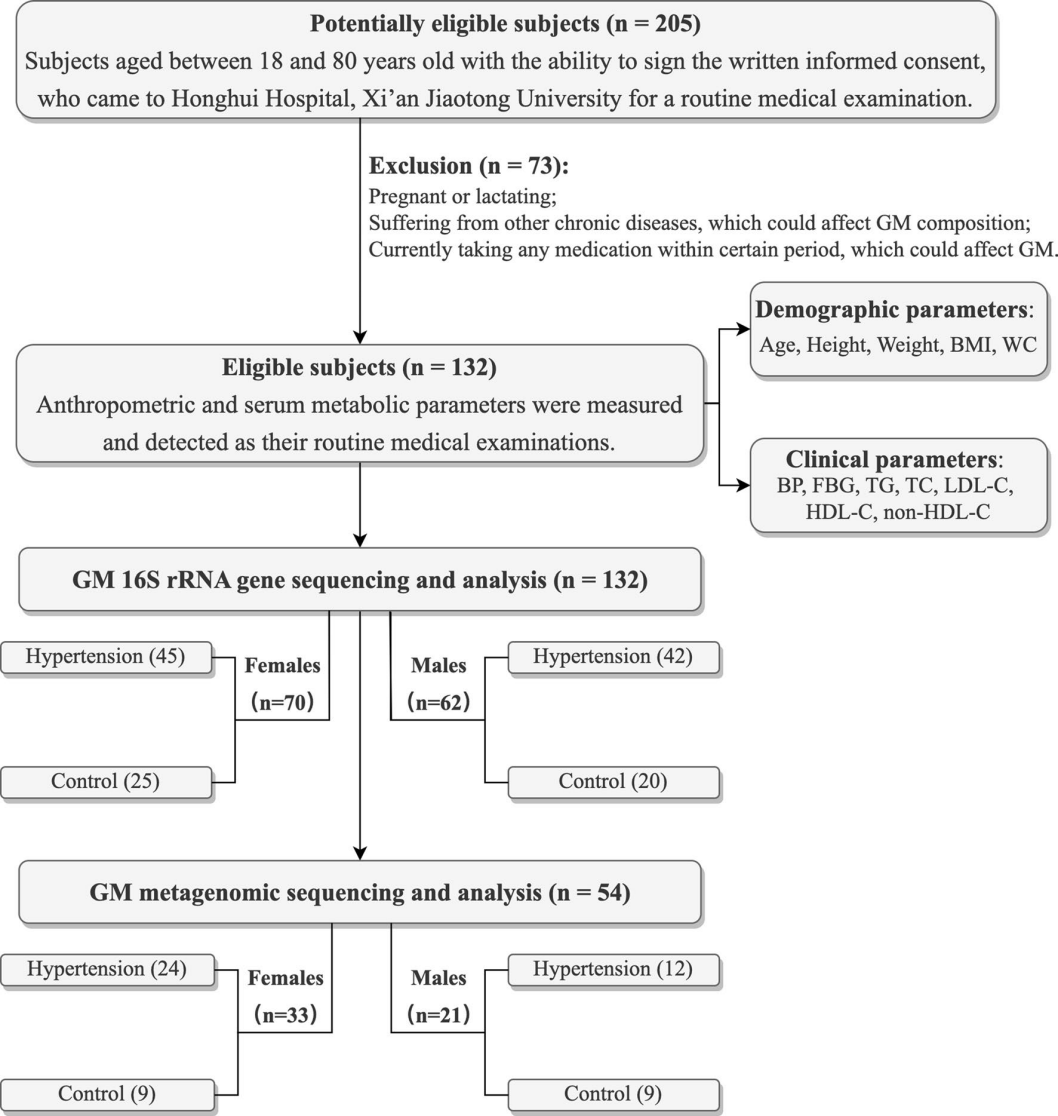
Study flow diagram. *BMI* body mass index; *WC* waist circumference; *BP* blood pressure; *FBG* fasting blood glucose; *TG* triglyceride; *TC* total cholesterol; *LDL-C* low-density lipoprotein cholesterol; *HDL-C* high-density lipoprotein cholesterol; *non-HDL-C* non-high-density lipoprotein cholesterol; *GM* gut microbiota

This study was approved by the Ethics Committee of Honghui Hospital, Xi’an Jiaotong University (Protocol Number: 201801022, approved January 8th, 2018), and all subjects provided written informed consent before enrollment. HTN was diagnosed if systolic BP (SBP) ≥ 130 mmHg and/or diastolic BP (DBP) ≥ 80 mmHg; subjects with normal BP (SBP < 120 mmHg and DBP < 80 mmHg) served as controls [26, 27]. The study flow is shown in Fig. 1.

### Measurements of demographic and clinical parameters

On their first visit, all medical information was recorded for the recruited subjects. Body weight and height were measured without shoes and with light clothing to the nearest 0.1 cm or to the nearest 0.1 kg, respectively. Body mass index (BMI) was calculated as weight in kilograms divided by the square of height in meters. Waist circumference (WC) was measured midway between the lower rib margin and the iliac crest in the standing position with a non-expandable tape to the nearest 0.1 cm. BP was assessed using a medical electronic sphygmomanometer (OMRON HEM-7130 professional portable blood pressure monitor, OMRON, Dalian, China) on the left arm positioned at heart level in a seated position with palm face up. The subjects were required to rest for at least 3 ~ 5 min before BP measurement, and BP was measured in duplicate at a 1 ~ 2-min interval. The average of the 2 readings was calculated. Mean arterial pressure (MAP) was calculated using the formula [(2 × diastolic BP) + systolic BP]/3 [16].

Peripheral venous blood samples were obtained in the morning after an overnight (at least 8 h) fast. Levels of fasting plasma glucose (FPG), serum triglyceride (TG), total cholesterol (TC), low-density lipoprotein cholesterol (LDL-C), high-density lipoprotein cholesterol (HDL-C) and non-high-density lipoprotein cholesterol (non-HDL-C) were measured using an automatic biochemical analyzer (Cobas c701, Roche, Mannheim, Germany). All instruments were calibrated regularly.

### Fecal sample collection and DNA extraction

We followed the standard protocols of fecal sample collection and processing [28]. Briefly, fresh fecal samples were collected from each participant at home, and temporarily stored in foam boxes with frozen cold packs. After immediate transportation to the Clinical Laboratory at Honghui Hospital, Xi’an Jiaotong University within 6 h, fecal samples were stored at − 80 °C until further processing.

Samples were sent in cold-chain with dry ice to Novogene Co., Led. (Beijing, China) for subsequent procedures. Genomic DNA was extracted from feces using the QIAamp Fast DNA Stool Mini Kit (Qiagen, Hilden, Germany) according to the manufacturer’s instructions. After the evaluation of genomic DNA concentration and quality, DNA samples, greater than 1 µg and with an OD value between 1.8 ~ 2.0, were qualified for further analysis.

### 16S rRNA gene amplification, sequencing and analysis

The V3 ~ V4 hypervariable regions of the 16S rRNA gene were amplified using specific primers (338F: 5ʹ-ACTCCTACGGGAGGCAGCAG-3ʹ; 806R: 5ʹ-GGACTACHVGGGTWTCTAAT-3ʹ) with barcodes, and all PCRs were performed with Phusion^®^ High Fidelity PCR Master Mix (New England Biolabs, Ipswich, MA, USA). After mixing the PCR products, the mixture was purified using a GeneJET™ Gel Extraction Kit (Thermo Scientific, Waltham, MA, USA). Sequencing libraries were generated using the NEB Next^®^ Ultra™ DNA Library Prep Kit for Illumina (New England Biolabs, Ipswich, MA, USA) following the manufacturer’s recommendations, and library quality was assessed on the Qubit^®^ 2.0 Fluorometer (Thermo Scientific, Waltham, MA, USA). Finally, the prepared libraries were sequenced on an Illumina HiSeq platform (Illumina NovaSeq 6000, PE150, Illumina, San Diego, CA, USA).

UPARSE software (Uparse v7.0.1001, http://drive5.com/uparse/) [29] and Quantitative Insights Into Microbial Ecology (QIIME) software (v1.7.0) [30] were introduced for sequencing analysis. Acquired high-quality clean reads with ≥ 97% similarity were de novo clustered into the same operational taxonomic units (OTUs), and the representative sequence for each OTU was screened and used to annotate taxonomic information based on the RDP classifier [31]. After OTUs with annotation were produced, microbial diversity was assessed using QIIME software (v1.7.0) [30]. Alpha-diversity analysis was performed based on 4 indices, including Shannon, Chao1, Simpson and abundance coverage-based estimator (ACE). Beta-diversity of GM composition was estimated using the unweighted UniFrac method to calculate the distances between samples, and then visualized by principal coordinates analysis (PCoA). Linear discriminant analysis (LDA) effect size (LEfSe) algorithm with an LDA score threshold of 2 (on a log10 scale) was applied to identify the enriched and significant bacteria in each group, with a *P* value < 0.05.

### Metagenomic sequencing and analysis

After DNA extraction and quality control, a total of 1 µg DNA per sample was used for library preparation. Sequencing libraries were generated using the NEBNext^®^ Ultra™ DNA Library Prep Kit (New England Biolabs, Ipswich, MA, USA) following the manufacturer’s recommendations. Briefly, the qualified DNA was fragmented to a size of 350 bp by sonication, and the acquired DNA fragments were end-polished, A-tailed, and ligated with the full-length adaptor for Illumina sequencing. Then PCR amplification was performed, and PCR products were purified using the AMPure XP system. Then, libraries were initially quantified using a Qubit^®^ 2.0 Fluorometer (Thermo Scientific, Waltham, MA, USA) and diluted to 2 ng/µL. Finally, libraries were analyzed using an Agilent2100 Bioanalyzer for size distribution, and then quantified using real-time PCR to ensure the quality (effective concentration > 3 nM). After clustering, the prepared libraries were sequenced on an Illumina HiSeq platform (Illumina NovaSeq 6000, PE250, Illumina, San Diego, CA, USA).

Short Oligonucleotide Analysis Package 2 (SOAP2) software (v2.04, http://soap.genomics.org.cn/soapdenovo.html) and Bowtie2.2.4 software were used for raw data processing, and the remaining Scaftigs were used for subsequent analysis. Genes were predicted on Scaftigs (≥ 500 bp) using MetaGeneMark (prokaryotic GeneMark.hmm v2.10), and a non-redundant gene catalog was constructed with CD-HIT (v4.5.8) software. For information on the abundance of genes, clean reads were realigned to the gene catalog (Unigenes) using Bowtie2.2.4 software. Genes with more than 2 mapped reads were deemed to be present in a sample [32]. The abundance of genes was calculated by counting the number of reads and normalizing based on gene length. Unigenes were aligned to the KEGG database (Release 73.1, Version: 2018.01, with animal and plant genes removed) for gene functional annotation using DIAMOND software (version 0.9.9), and GM functions were evaluated and compared across groups in the present study.

### Statistical analysis

Statistical analyses and figure constructions were conducted using the SPSS PASW v23 (IBM SPSS Inc., Chicago, IL), R platform v4.0.2 (R Foundation, Vienna, Austria), GraphPad Prism v5.01 (GraphPad Software Inc., San Diego, CA, USA) and MedCalc v19.0.4 (MedCalc Software Bvba, Ostend, Belgium) software. Quantitative variables are presented as mean ± standard deviation (SD), and the normal distribution of quantitative variables was assessed by the Shapiro-Wilk test. The t-test was used for comparisons of the demographic and clinical parameters between groups, depending on the homogeneity of variance. The comparisons of GM diversities between groups were assessed using the Wilcoxon rank-sum test. The top 10 GM taxa in each level (phylum, class, order, family, genus and species) sorted by higher relative abundances were identified, and the significant differences between groups were assessed using the Wilcoxon rank-sum test. The “PerformanceAnalytics” package in R was utilized for Spearman’s correlation analysis between any two demographic or clinical parameters of the subjects. In addition, Spearman’s correlation test was used to analyze the correlations between GM composition/KEGG functions and BP levels. A *P* value < 0.05 was considered statistically significant, whereas a *P* value between 0.05 and 0.1 was considered a tendency. Receiver operator characteristic (ROC) curve analysis was performed, and GM functions with an area under the curve (AUC) ≥ 0.700 were selected for presentation.

## Results

### Demographic and clinical characteristics of the study subjects

**Fig. 2:**
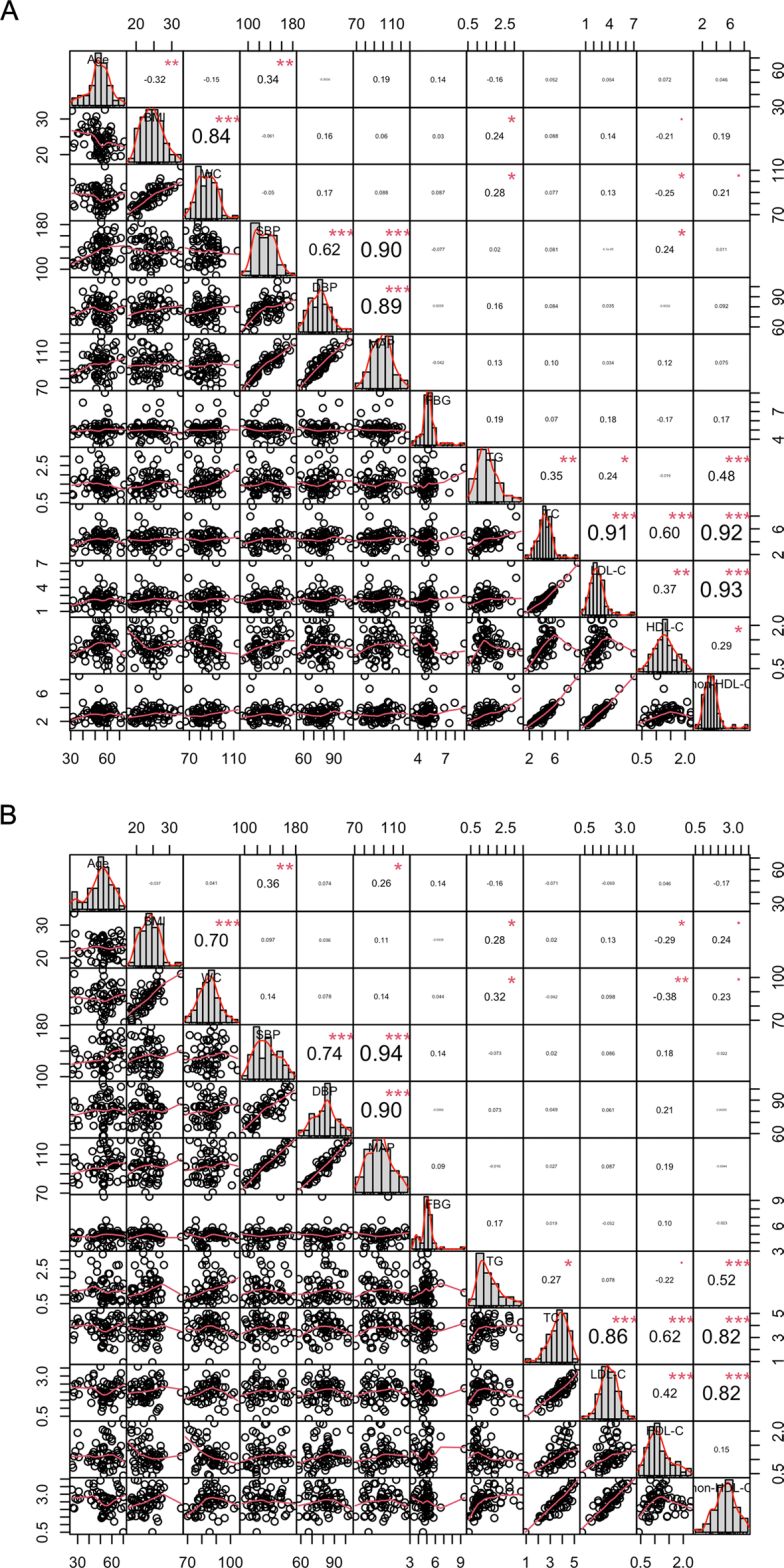
Correlation analysis between demographic and clinical characteristics of the study subjects. Correlation analysis between demographic and clinical characteristics of the enrolled females (**A**) and males (**B**), respectively. Bar plots present the distribution of each parameter, and the scatter plots show the distribution for each two parameters. The numbers in this figure indicate the correlation coefficients of each two parameters by Spearman method in R, and the red dots and asterisks indicate the degrees of statistical significances, ^•^*P* < 0.1, ^*^P < 0.05, ^**^P < 0.01, ^***^P < 0.001

As described above, 87 HTN subjects and 45 controls were finally recruited in this study. Table 1 shows the demographic and clinical characteristics of the study subjects grouped by sex and BP levels. With the exception of SBP, DBP and MAP (*P* < 0.05), the other parameters showed no significances between HTN and control females, as well as HTN and control males. Figure 2 shows the correlations between any two characteristics of the enrolled females and males.

**Table 1:** Demographic and clinical characteristics of the enrolled subjects for GM 16S rRNA gene sequencing in this study

Variables	Female		*P* value	Male		*P* value
	HTN	control		HTN	control	
Number	45	25	–	42	20	–
Age (year)	55 ± 7	51 ± 11	0.064	54 ± 11	48 ± 12	0.059
Height (cm)	153.2 ± 6.7	152.7 ± 7.7	0.800	165.7 ± 6.4	164.6 ± 8.5	0.564
Weight (kg)	56.9 ± 9.8	55.7 ± 9.8	0.626	64.3 ± 9.6	61.5 ± 8.4	0.262
BMI (kg/m2)	24.2 ± 3.6	23.8 ± 3.3	0.646	23.4 ± 3.2	22.7 ± 2.9	0.411
WC (cm)	85.9 ± 9.2	84.6 ± 8.5	0.554	86.0 ± 8.4	83.3 ± 7.2	0.213
SBP (mmHg)	145 ± 15	110 ± 7	0.000*	142 ± 17	112 ± 7	0.000*
DBP (mmHg)	82 ± 9	68 ± 6	0.000*	86 ± 8	69 ± 5	0.000*
MAP (mmHg)	103 ± 9	82 ± 6	0.000*	105 ± 9	83 ± 5	0.000*
FPG (mmol/L)	5.06 ± 0.94	5.09 ± 0.78	0.889	4.82 ± 1.00	4.81 ± 0.65	0.948
TG (mmol/L)	1.61 ± 0.55	1.51 ± 0.59	0.460	1.40 ± 0.70	1.27 ± 0.77	0.510
TC (mmol/L)	4.40 ± 1.07	4.36 ± 1.62	0.909	3.75 ± 0.70	3.53 ± 1.14	0.428
LDL-C (mmol/L)	2.49 ± 0.78	2.60 ± 1.31	0.653	2.07 ± 0.56	1.99 ± 0.72	0.630
HDL-C (mmol/L)	1.37 ± 0.44	1.20 ± 0.40	0.115	1.22 ± 0.40	1.07 ± 0.47	0.179
non-HDL-C (mmol/L)	3.03 ± 0.84	3.16 ± 1.56	0.646	2.53 ± 0.59	2.46 ± 0.91	0.774

Data are presented as mean ± standard deviation (SD)

*BMI* body mass index, *WC* waist circumference, *SBP* systolic blood pressure, *DBP* diastolic blood pressure, *MAP* mean arterial pressure, *FBG* fasting plasma glucose, *TG* triglyceride, *TC* total cholesterol, *LDL-C* low-density lipoprotein cholesterol, *HDL-C* high-density lipoprotein cholesterol, *non-HDL-C* non-high-density lipoprotein cholesterol

*P* values are from t-test depending on the homogeneity of variance, ^*^P < 0.05

### 16S rRNA gene sequencing analysis of gut microbiota in the study subjects

**Fig. 3:**
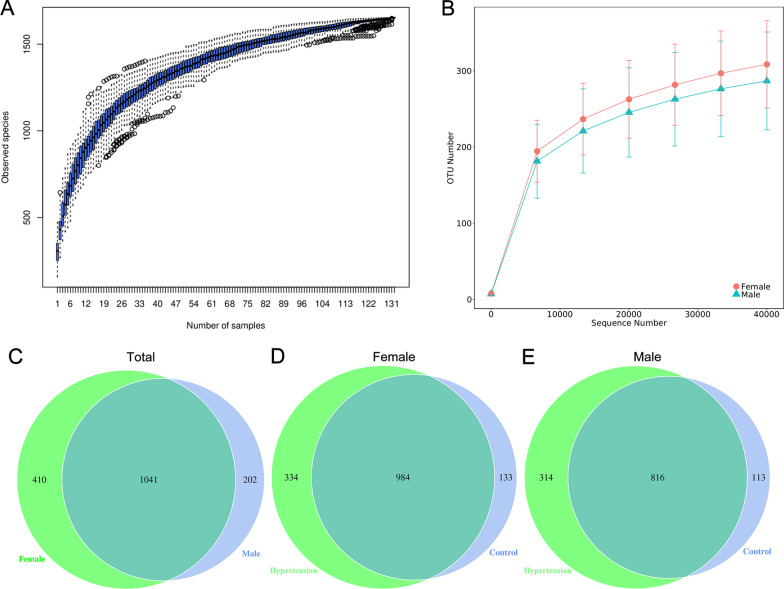
The basic information of the 16S rRNA gene sequencing. The species accumulation curve of gut microbiota detected in HTN and control subjects in this study (**A**). The line indicates the averaged accumulated increase of detected OTUs vs. number of samples. The box-plots show the 25*th*, 50*th* and 75*th* percentile at each sample size. The rarefaction curve of the number of sequence reads and their corresponding number of OTUs in females and males in this study (**B**). Venn diagrams of observed OTU numbers in different comparison groups: females vs. males (**C**), HTN group vs. control group in females (**D**) and males (**E**), respectively. Every circle depicts the number of unique OTUs observed in one group. Overlapping OTUs shared by two groups are represented in the areas of intersection among corresponding circles

After 16S rRNA gene sequencing, the species accumulation curve of GM in Fig. 3A and the rarefaction curves in Fig. 3B showed a plateau of species richness, indicating that the sample numbers and sequencing depth herein covered enough information for the following analysis. Finally, we acquired a total of 1653 unique OTUs (ranging from 208 to 416 per sample), and please refer to Fig. 3C ~ 3E for details.

**Fig. 4:**
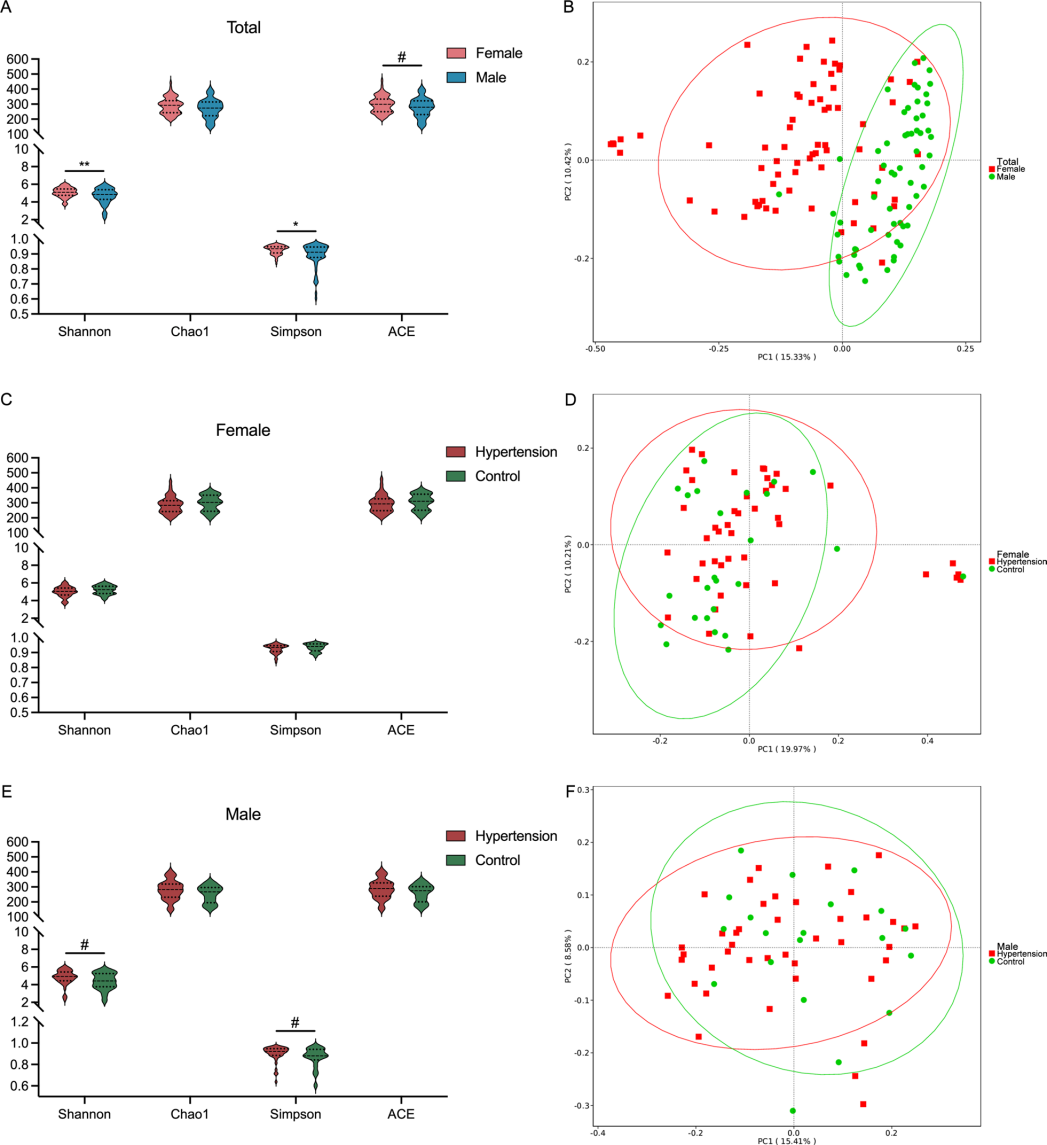
The diversity analysis of gut microbiota in the study subjects. Violin plots of α-diversity analysis in different comparison groups: females vs. males (**A**), HTN group vs. control group in females (**C**) and males (**E**), respectively. Each plot represents one index of the α-diversity distribution, including Shannon, Chao1, Simpson and ACE indices, for each comparison groups. Wilcoxon rank-sum test was used for the analysis of significant differences between different comparison groups. ^#^*P* < 0.1, ^*^P < 0.05, ^**^P < 0.01. Plots of principal coordinate analysis (PCoA) based on the OTU level in different comparison groups: females vs. males (**B**), HTN group vs. control group in females (**D**) and males (**F**), respectively. Each square/circle indicates one sample. The distance between samples represents the GM similarity or differences in the samples, and the PCoA analysis was conducted with unweighted UniFrac method

Initially, we evaluated the α-diversities of GM in females and males, which were significantly lower in males compared with females (Fig. 4A). Moreover, the PCoA model for β-diversity analysis revealed a segregation between females and males (Fig. 4B). Therefore, the study subjects should be divided into female and male groups in the following analysis. Then, we conducted the α- and β-diversity analyses between the HTN and control subjects in females and males, respectively. However, the differences of GM diversities were not significant between either HTN vs. control females (Fig. 4C, D), or HTN vs. control males (Fig. 4E, F).

**Fig. 5:**
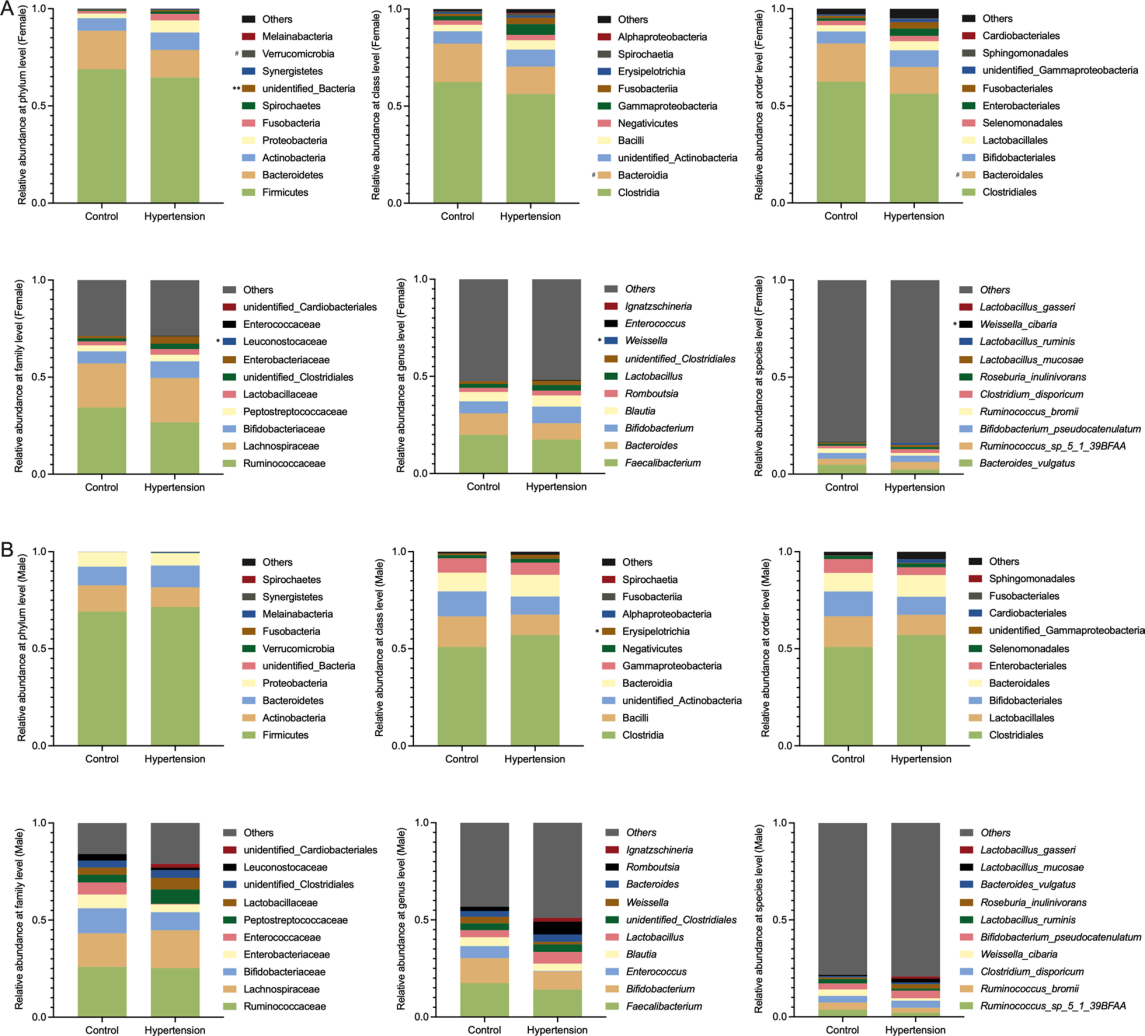
Relative abundances and comparative analysis of the taxonomic composition of gut microbiota in the study subjects. Relative abundances and comparative analysis of the taxonomic composition of gut microbiota in the enrolled females (**A**) and males (**B**), respectively. Bar plots show the relative abundances of the top 10 taxa at respective levels, including phyla, class, orders, family, genera and species, in HTN and control females and males. Each component of the cumulative bar chart indicates a phylum, a class, an order, a family, a genus or a species, respectively. The taxa with significant difference between groups are presented using Wilcoxon rank-sum test. ^#^*P* < 0.1, ^*^P < 0.05, ^**^P < 0.01

**Fig. 6:**
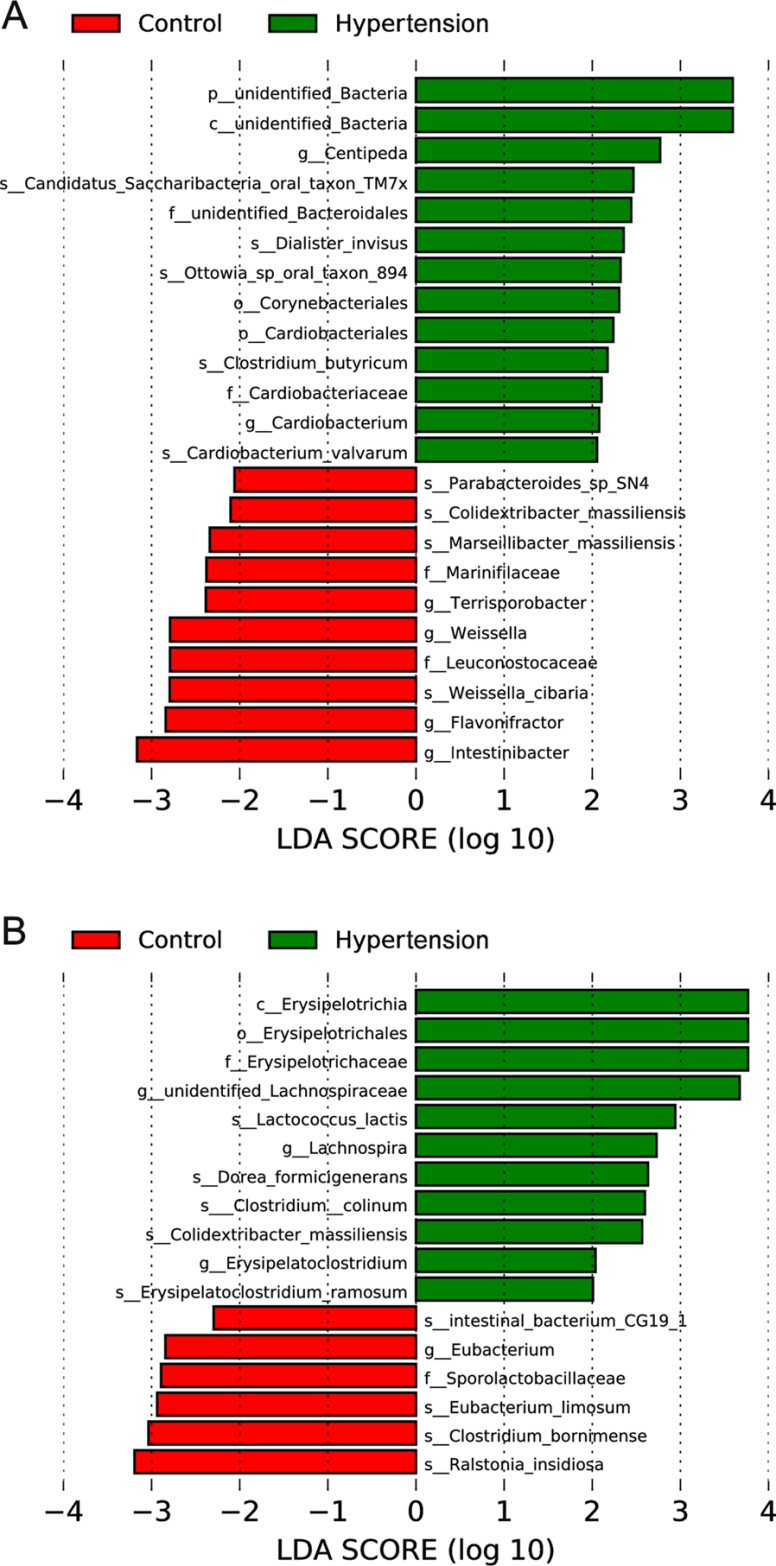
Linear discriminant analysis (LDA) effect size (LEfSe) analysis of gut microbiota taxa in the study subjects. LDA scores indicate differentially represented taxa in HTN and control groups in females (**A**) and males (**B**), respectively. The logarithmic threshold for discriminative features was set to 2.0

The differences of GM composition between HTN and control subjects were taxonomically evaluated at six different levels, including phylum, class, order, family, genus and species, and the top 10 GM taxa with higher relative abundances were summarized in each level (Fig. 5). Consistent with previous results, the GM taxa were mostly included in four predominant phyla, which were Firmicutes, Bacteroidetes, Actinobacteria and Proteobacteria. The relative abundances of Firmicutes, Bacteroidetes, Clostridia, Bacteroidia, Clostridiales, Bacteroidales, Ruminococcaceae, Lachnospiraceae and *Faecalibacterium* were greater than 0.100 at respective taxonomic levels in HTN and control females. Of note, the unidentified_Bacteria abundance was greater in HTN females, whereas the relative abundances of Bacteroidia, Bacteroidales, Leuconostocaceae, *Weissella* and *Weissella_cibaria* were lower in HTN females compared with control females (Fig. 5A). LEfSe analysis indicated that unidentified_Bacteria was enriched in HTN females; while, Leuconostocaceae, *Weissella* and *Weissella_cibaria* were enriched in control females (Fig. 6A). In contrast, no significant differences of GM composition were noted between HTN and control males except for Erysipelotrichia, which was enriched in HTN males (Figs. 5B and 6B). Furthermore, the correlations between GM taxa and SBP/DBP/MAP levels were investigated in females and males, respectively, and data were showed in Additional file 1: Tables S1 and S2 in the Additional files.

### Metagenomic sequencing analysis of gut microbiota

After the preliminary analysis based on the 16S rRNA gene sequencing, we conducted further analysis of GM functions by performing matagenomic sequencing. A total of 36 HTN subjects (24 females and 12 males) and 18 controls (9 females and 9 males) were randomly selected for metagenomic analysis. Their demographic and clinical characteristics were demonstrated in Table 2 grouped by sex and BP levels.

**Table 2:** Demographic and clinical characteristics of the enrolled subjects for GM metagenomic sequencing in this study

Variables	Female		*P* value	Male		*P* value
	HTN	control		HTN	control	
Number	24	9	–	12	9	–
Age (year)	54 ± 7	54 ± 7	0.925	57 ± 6	52 ± 4	0.040*
Height (cm)	153.1 ± 6.1	153.7 ± 7.4	0.822	167.2 ± 6.5	160.9 ± 7.3	0.052
Weight (kg)	58.7 ± 10.1	55.2 ± 11.0	0.395	66.3 ± 9.8	58.9 ± 11.2	0.126
BMI (kg/m2)	25.0 ± 3.7	23.1 ± 2.9	0.190	23.7 ± 2.9	22.7 ± 3.2	0.453
WC (cm)	88.5 ± 9.8	85.1 ± 11.4	0.405	89.2 ± 9.5	82.4 ± 9.3	0.118
SBP (mmHg)	145 ± 17	105 ± 8	0.000*	136 ± 12	112 ± 6	0.000*
DBP (mmHg)	83 ± 9	65 ± 7	0.000*	83 ± 6	69 ± 6	0.000*
MAP (mmHg)	103 ± 9	78 ± 5	0.000*	100 ± 5	83 ± 5	0.000*
FPG (mmol/L)	5.24 ± 1.15	5.38 ± 0.74	0.740	4.58 ± 0.67	4.84 ± 0.88	0.447
TG (mmol/L)	1.71 ± 0.62	1.68 ± 0.83	0.898	1.40 ± 0.78	1.05 ± 0.55	0.255
TC (mmol/L)	4.35 ± 1.14	5.39 ± 1.99 ±	0.068	3.51 ± 0.95	3.62 ± 0.86	0.780
LDL-C (mmol/L)	2.45 ± 0.82	3.36 ± 1.70	0.155	1.88 ± 0.78	2.01 ± 0.63	0.702
HDL-C (mmol/L)	1.26 ± 0.42	1.34 ± 0.46	0.662	1.11 ± 0.44	1.22 ± 0.48	0.559
non-HDL-C (mmol/L)	3.09 ± 0.90	4.05 ± 2.16	0.226	2.40 ± 0.69	2.40 ± 0.77	0.987

Data are presented as mean ± standard deviation (SD)

*BMI* body mass index, *WC* waist circumference, *SBP* systolic blood pressure, *DBP* diastolic blood pressure, *MAP* mean arterial pressure, *FBG* fasting plasma glucose, *TG* triglyceride, *TC* total cholesterol, *LDL-C* low-density lipoprotein cholesterol, *HDL-C* high-density lipoprotein cholesterol, *non-HDL-C* non-high-density lipoprotein cholesterol

*P* values are from t-test depending on the homogeneity of variance, ^*^P < 0.05

**Fig. 7:**
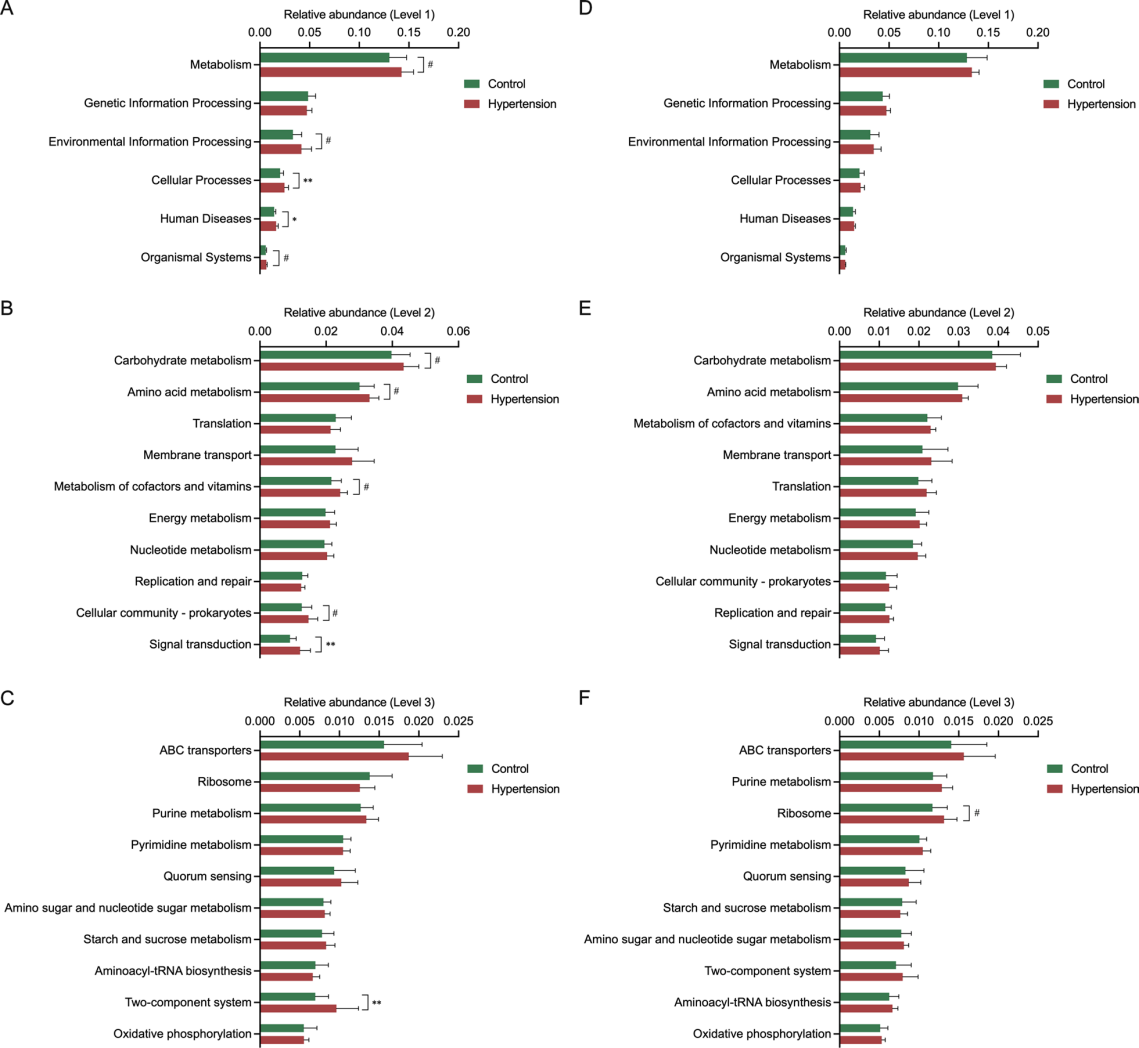
Relative abundances and comparative analysis of the annotated KEGG functions of gut microbiota in the study subjects. Relative abundances and comparative analysis of the annotated KEGG functions of GM at three levels in HTN and control groups in females (**A–C**) and males (**D–F**), respectively. The KEGG functions with significant differences between groups are presented with Wilcoxon rank-sum test. ^#^*P* < 0.1, ^*^P < 0.05, ^**^P < 0.01

After GM gene and functional annotation aligned to the KEGG database, the functional changes in the microbial community were evaluated. GM functions showed different patterns between HTN and control subjects in females and males, as presented in Fig. 7. Our results revealed that Cellular Processes and Human Diseases represented the enrichment in Level 1 KEGG functions in HTN females (*P* < 0.05), as well as Metabolism, Environmental Information Processing and Organismal System (*P* < 0.1, Fig. 7A). In addition, the relative abundance of Signal transduction in Level 2 and Two-component system in Level 3 were increased in HTN females compared with controls (*P* < 0.05, Fig. 7B, C). Please refer to Fig. 7D ~ 7 F for detailed information about male subjects. Notably, the relative abundances of Metabolism, Environmental Information Processing, Cellular Processes, Human Diseases, Carbohydrate metabolism, Amino acid metabolism, Membrane transport, Cellular community – prokaryotes, ABC transporters and Quorum Sensing were enriched in females compared to males (*P* < 0.05); please refer to Additional file 1: Figure S1 in the additional files for details. These data further supported our previous strategy of separation of female and male subjects in GM analysis.

**Fig. 8:**
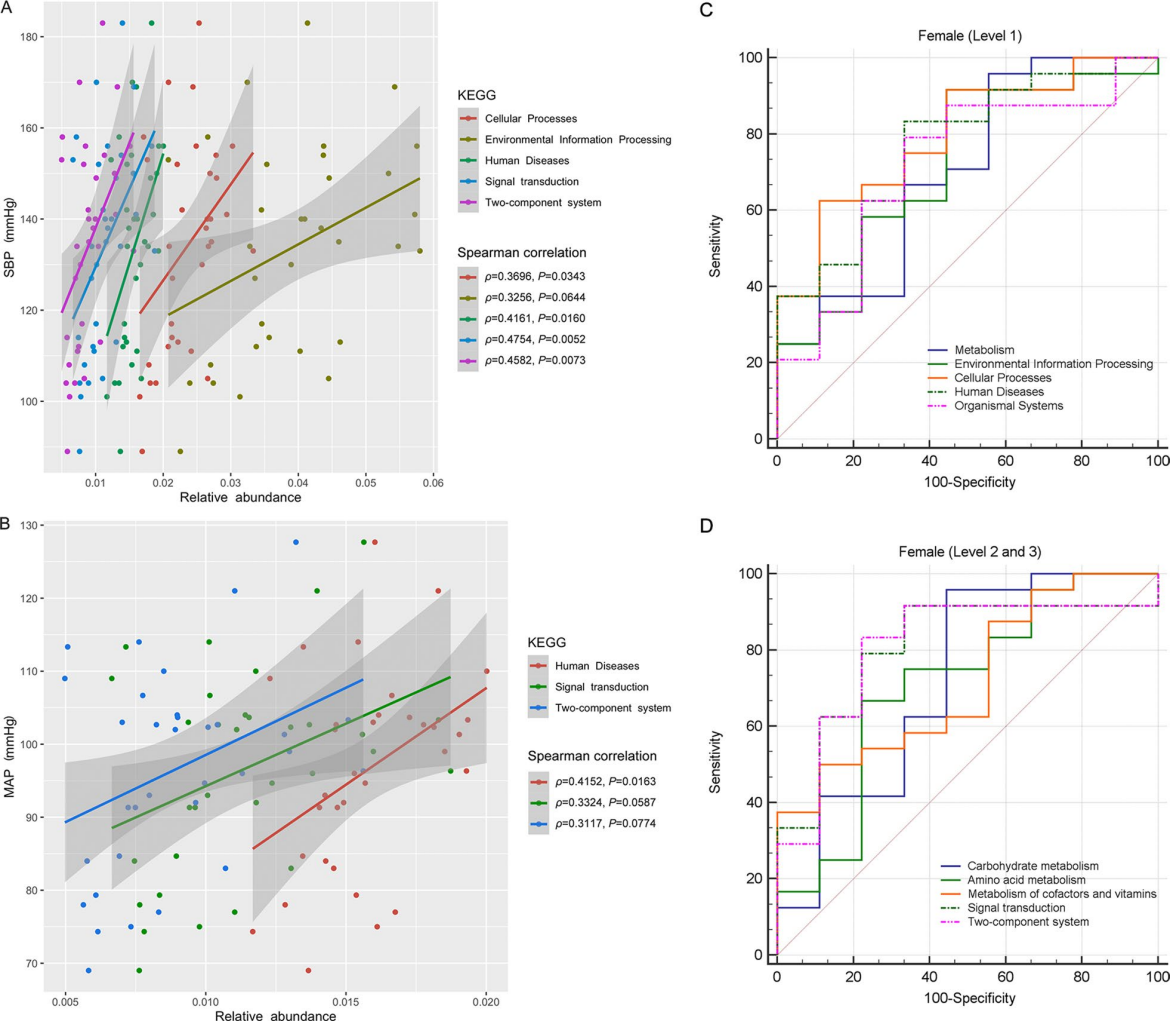
Exploration of the altered KEGG functions of gut microbiota in the enrolled HTN females. Correlations of altered KEGG functions of gut microbiota with SBP (**A**) and MBP (**B**) levels in the enrolled females. The horizontal axis represents the relative abundances of KEGG functions, and the vertical axis represents different BP levels. Data was analyzed and plotted with Spearman method in R. Receiver operator characteristic (ROC) curve analysis of identification of HTN females from controls based on KEGG functions of gut microbiota (**C**, **D**)

To explore the implications of altered GM functions in HTN pathogenesis, Spearman’s correlation analysis was introduced to evaluate the correlations between GM functions and BP levels. Significantly positive correlations of Cellular Processes, Human Diseases, Signal transduction and Two-component system with SBP were found in females (*P* < 0.05, Fig. 8A). Besides, the correlation of Human Diseases with MAP was also noted (*P* < 0.05, Fig. 8B). To further explore the altered GM functions that could facilitate the identification of HTN subjects, ROC curve analysis was introduced herein (Fig. 8C, D). The AUCs of GM functions in Level 1 that could differentiate HTN females from controls included Cellular Processes (0.796, 95% CI 0.620 ~ 0.916), Human Diseases (0.773, 95% CI 0.595 ~ 0.900), Environmental Information Processing (0.718, 95% CI 0.534 ~ 0.860), Organismal Systems (0.718, 95% CI 0.534 ~ 0.860) and Metabolism (0.704, 95% CI 0.520 ~ 0.849). Besides, Signal transduction (0.806, 95% CI 0.631 ~ 0.922), Two-component system (0.806, 95% CI 0.631 ~ 0.922), Carbohydrate metabolism (0.722, 95% CI 0.539 ~ 0.863), Metabolism of cofactors and vitamins (0.718, 95% CI 0.534 ~ 0.860), and Amino acid metabolism (0.708, 95% CI 0.525 ~ 0.853) in Level 2 and 3 could effectively distinguish HTN females from controls.

## Discussion

Hypertension (HTN) is a multifactorial and complicated condition [3, 4], and growing evidence suggests a novel role of GM in HTN onset and progression [16, 33, 34]. The adult GM consists trillions of microorganisms dominated by phyla Firmicutes, Bacteroidetes, Actinobacteria and Proteobacteria, and maintains the gut immunity and whole-body homeostasis [35]; dysbiosis or imbalance in the GM community may have detrimental effects on health [36]. Several demographic factors, such as age, BMI, sex, ethnicity and culture, geographic location and socioeconomic environment, could influence both GM and BP levels [2, 15, 21, 24, 37–39], and it is important to consider these confounding factors in GM-related studies on HTN. Although biological sex could shape the host GM [40–42], available data directly linking GM to HTN based on sex differences are limited [21]. Therefore, residents with similar dietary habits living in Shaanxi Province, China, were recruited to minimize the regional differences of GM composition in this study; and the differences of GM characteristics between female and male subjects were analyzed. Of note, an obvious segregation of GM diversity between females and males was found, supporting a respective analysis for female and male subjects in the subsequent GM analysis.

GM has been implicated in the pathogenesis of HTN by means of influencing sodium intake, production of certain metabolites, low-grade inflammation, etc. [37, 43]. Epidemiological data have linked salt and fiber intake with changes in BP levels, suggesting a connection between gut and HTN [3, 4, 44–47]. High sodium intake could reduce the relative abundances of certain beneficial taxa such as *Lactobacillus spp*, and GM-produced metabolites may also influence sodium absorption [48]. Besides, an increased consumption of dietary fiber, which could modulate GM as well as be fermented by GM, has been indicated to decrease BP levels [45, 46, 48, 49]. The potent mechanism may involve the production of short-chain fatty acids (SCFAs), such as acetate, butyrate and propionate, which are mainly derived from the fermentation of indigestible carbohydrates and consumption of protein or peptide [15, 49–52]. GM-produced SCFAs are rapidly absorbed in the colon and taken up by the liver or enter the circulation, and serve as precursors or substrates in various physiological processes [53–55]. In rodent models, SCFAs were reported to bind to G-protein-coupled receptors involved in the regulation of vasoreactivity and BP levels [56–58]. The negative correlations between the abundances of butyrate-producing bacteria and BP levels have been observed in obese pregnant women [59], and fiber and acetate supplementation could improve GM dysbiosis and increase the relative abundances of certain bacteria that may play a protective role in HTN [49]. In summary, SCFAs and a range of SCFA-producing taxa could play potent roles in maintaining GM homeostasis and BP levels [33, 60–62]. It is known that Bacteroidetes phylum members could produce high levels of acetate and propionate, whereas certain species in Firmicutes may produce high amounts of butyrate [63–65]. Consistent with these data, our results showed that the relative abundances of Bacteroidia and Bacteroidales were greater than 0.10 at respective taxonomic levels, and were lower in HTN females compared with controls. Besides, Leuconostocaceae, *Weissella* and *Weissella_cibaria* were also enriched in control females. As Firmicutes phylum members, Leuconostocaceae, *Weissella* and *Weissella_cibaria* are producers of SCFAs, and *Weissella cibaria* may have antihypertensive and antioxidant effects in spontaneously hypertensive rats models [66, 67], indicating their potential for HTN prevention and further supporting our results herein.

Ruminococcaceae is involved in intestinal epithelium maintenance as it is inversely correlated with intestinal permeability [68–70], and its abundance was found diminished in elderly HTN patients [71]. Lower abundance of *Ruminococcus* may associate with HTN [72–74], and a particular reduction in *Ruminococcus* was also found in SD rats with minocycline-induced programmed HTN [75]. However, Kim SR et al. reported a higher abundance of *Ruminococcus torques* in HTN, which was significantly associated with SBP [15]; while, Dan et al. showed increased Ruminococcaceae and decreased *Ruminococcus* in HTN subjects [76]. In this study, *Ruminococcus_bromii*, as a member of Firmicutes and *Ruminococcus*, was positively correlate with DBP. In other words, certain inconsistent results exist, which may be due to variations in genetics, sex, diet and lifestyle, geographical differences or other unknown factors [37]. Moreover, these data implicated a more important role of individual GM taxon than phyla in BP regulation [21].

GM-related studies indicate that GM could exert potential influences on various diseases [35, 77–80], and certain GM characteristics could be utilized as non-invasive biomarkers for early diagnosis in clinical practice [81, 82]. As one of the most prevalent CVDs and a leading risk factor of other CVDs, HTN is certainly accompanied by GM alterations [83–85]. Exploration of the altered KEGG functions of GM in HTN may help illustrate its functional roles in HTN pathogenesis, and might provide a new perspective on the interpretation of HTN and additional auxiliary diagnosis in the future [86]. In this study, we randomly selected HTN and control subjects from the recruited females and males, who also lacked significant differences in the majority of these demographic and clinical characteristics, for metagenomic sequencing analysis. The relative abundances of Human Diseases, Signal transduction and Two-component system were increased in HTN females compared with controls, positively correlated with increased SBP and MAP levels, and contributed to the effective identification of HTN females from controls. Besides, our data indicated that the altered GM functions differed between HTN females and males compared to respective controls, further supporting the notion that females and males should be separately analyzed in GM-related analysis. In terms of sex differences in this study, we found higher α-diversities of GM in females, as well as an obvious segregation in β-diversity between females and males, and the GM composition and functions were also different between female and male subjects, which could be contributed by genetic and epigenetic factors, sex steroid milieu, gonadal status, dietary and lifestyle-related factors, etc. [40, 87–89]. Specifically, previous data indicated that the primary sex steroid hormones, such as estrogen, progesterone and testosterone, may participate in the regulation of GM diversity, composition and function [38, 40, 89]. For example, it was reported that estrogen could promote the growth of certain beneficial bacteria such as *Lactobacillus* and *Bifidobacterium*, which were also considered to improve the cardiovascular health [18, 89–91]. Moreover, sex differences also exist in lifestyle-related factors, such as dietary preferences, sex*diet interactions and physical activity levels, which may influence the GM characteristics and HTN onset and progression differently in females and males [18, 19, 42]. The present study was conducted in northwestern China, and regional variations in diet, lifestyle and environmental factors may potentially contribute to the observed differences in GM characteristics between females and males. It is crucial to note that the specific mechanisms underlying the observed differences herein are multifactorial and complicated, and may not be fully elucidated. Further researches are needed to better reveal the potential causes for the observed sex-based differences in GM, to elucidate the underlying mechanisms, and to facilitate understanding of the implications of GM for health and diseases, including HTN.

Although our data revealed certain GM changes in HTN subjects, it has several limitations. First, this investigation was conducted with a limited sample size due to the practical limitations of data availability. Second, the subjects were recruited in a single hospital and were grouped based on BP levels alone. Third, BP measurement in clinic is used for HTN diagnosis [92] rather than office BP monitoring [9]. Nevertheless, certain confounding factors were taken into consideration herein, such as genetics, geography, sex and treatment-naive recruitment, thus our data were reliable despite some other or unknown factors. Furthermore, evidence directly linking GM and sex differences in BP regulation is rather limited, and the role of GM in sex-dependent HTN is only hypothesized [2, 21, 22, 42]. Our data could provide evidence of fecal GM characteristics in HTN females and males, respectively, which might fill in the gap to a certain degree. However, this is only a cross-sectional clinical study in China, and it is infeasible to draw any definite conclusions about the causal relationships between GM and HTN based on sex differences. Issues regarding the differences in GM profiles of HTN females and males remain to be addressed in future studies [72], and detailed evaluations are needed in larger numbers of treatment-naive HTN patients.

## Conclusions

In conclusion, the pathogenesis of HTN is multifactorial and complicated, and evidence suggests that GM may play a novel role in HTN onset and progression. This study provide the first evidence of GM characteristics and alterations in HTN females and males, respectively, in northwestern China, further supporting the theory that GM dysbiosis underlies HTN pathogenesis. Future studies are needed to elucidate the underlying mechanisms and potential therapeutic interventions targeting GM for HTN prevention and management [14].

## Supplementary Information

Additional file 1: Table S1  Correlations between gut microbiota taxa and blood pressure levels in female subjects. Table S2 Correlations between gut microbiota taxa and blood pressure levels in male subjects. Fig. S1 Relative abundances and comparative analysis of the annotated KEGG functions of gut microbiota in female and male subjects. The KEGG functions with significant differences between groups are presented by Wilcoxon rank-sum test. #P < 0.1, *P < 0.05, **P < 0.01.

## Data Availability

The data generated and analyzed during the current study are available from the corresponding authors on reasonable request.

## Abbreviations

CVDs: Cardiovascular diseases
HTN: Hypertension
BP: Blood pressure
GM: Gut microbiota
SBP: Systolic blood pressure
DBP: Diastolic blood pressure
BMI: Body mass index
WC: Waist circumference
MAP: Mean arterial pressure
FPG: Fasting plasma glucose
TG: Triglyceride
TC: Total cholesterol
LDL-C: Low-density lipoprotein cholesterol
HDL-C: High-density lipoprotein cholesterol
non-HDL-C: Non-high-density lipoprotein cholesterol
OTUs: Operational taxonomic units
ACE: Abundance coverage-based estimator
PCoA: Principal coordinates analysis
LDA: Linear discriminant analysis
LEfSe: Linear discriminant analysis effect size
SD: Standard deviation
ROC: Receiver operator characteristic
AUC: Area under the curve
