# Supplementary material for: Alterations of gut microbiota are associated with blood pressure: a cross-sectional clinical trial in Northwestern China
Source: J Transl Med. 2023 Jun 30;21:429. doi: 10.1186/s12967-023-04176-6 (PMC10311887; doi:10.1186/s12967-023-04176-6)
Supplement: Supplementary file 1 — Additional file 1: Table S1 Correlations between gut microbiota taxa and blood pressure levels in female subjects. Table S2 Correlations between gut microbiota taxa and blood pressure levels in male subjects. Fig. S1 Relative abundances and comparative analysis of the annotated KEGG functions of gut microbiota in female and male subjects. The KEGG functions with significant differences between groups are presented by Wilcoxon rank-sum test. #P < 0.1, *P < 0.05, **P < 0.01. [file 12967_2023_4176_MOESM1_ESM.docx]

**Additional tables**

**Table S1** Correlations between gut microbiota taxa and blood pressure (SBP/DBP/MAP) levels in female subjects

| **Blood pressure** | **Gut microbiota taxa** | ***rho*** | ***P* value** |
| --- | --- | --- | --- |
| **SBP** | p_Bacteroidetes | -0.2179 | 0.0700 |
|  | p_unidentified_Bacteria | 0.3686 | 0.0017 |
|  | c_Bacilli | 0.2183 | 0.0694 |
|  | c_Negativicutes | -0.2130 | 0.0766 |
|  | o_Lactobacillales | 0.2079 | 0.0842 |
|  | o_Bacteroidales | -0.2309 | 0.0544 |
|  | o_Selenomonadales | -0.2161 | 0.0724 |
|  | f_Leuconostocaceae | -0.1987 | 0.0992 |
|  | g_Weissella | -0.2303 | 0.0551 |
|  | s_Weissella_cibaria | -0.2028 | 0.0922 |
|  | s_Lactobacillus_mucosae | 0.2139 | 0.0754 |
| **DBP** | p_Actinobacteria | 0.2878 | 0.0157 |
|  | c_unidentified_Actinobacteria | 0.2856 | 0.0165 |
|  | o_Bifidobacteriales | 0.2697 | 0.0239 |
|  | f_Peptostreptococcaceae | -0.2117 | 0.0785 |
|  | f_unidentified_Clostridiales | -0.2384 | 0.0469 |
|  | f_Bifidobacteriaceae | 0.2705 | 0.0235 |
|  | g_unidentified_Clostridiales | -0.2022 | 0.0932 |
|  | g_Bifidobacterium | 0.2693 | 0.0242 |
|  | s_Ruminococcus_bromii | 0.2378 | 0.0474 |
|  | s_Bifidobacterium_pseudocatenulatum | 0.2658 | 0.0262 |
| **MAP** | p_Bacteroidetes | -0.1987 | 0.0992 |
|  | p_Actinobacteria | 0.2821 | 0.0180 |
|  | p_unidentified_Bacteria | 0.2115 | 0.0788 |
|  | c_Bacteroidia | -0.2007 | 0.0957 |
|  | c_unidentified_Actinobacteria | 0.2794 | 0.0192 |
|  | o_Bacteroidales | -0.2056 | 0.0878 |
|  | o_Bifidobacteriales | 0.2633 | 0.0276 |
|  | f_Bifidobacteriaceae | 0.2634 | 0.0276 |
|  | g_Bifidobacterium | 0.2638 | 0.0274 |
|  | s_Lactobacillus_mucosae | 0.2322 | 0.0531 |
|  | s_Bifidobacterium_pseudocatenulatum | 0.2275 | 0.0582 |

p: phylum; c: class; o: order; f: family; g: genus; s: species.

**Table S2** Correlations between gut microbiota taxa and blood pressure (SBP/DBP/MAP) levels in male subjects

| **Blood pressure** | **Gut microbiota taxa** | ***rho*** | ***P* value** |
| --- | --- | --- | --- |
| **DBP** | g_Blautia | 0.2457 | 0.0542 |
|  | s_Ruminococcus_sp_5_1_39BFAA | 0.2149 | 0.0935 |

p: phylum; c: class; o: order; f: family; g: genus; s: species.

**Additional figures**


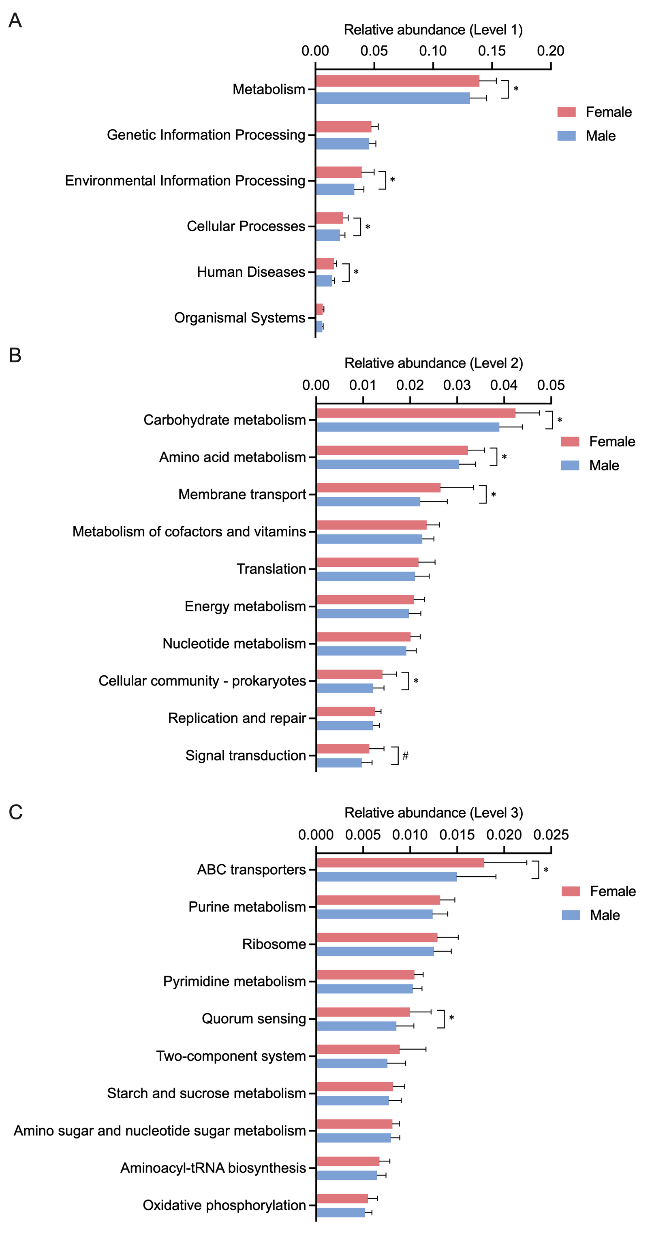


**Fig. S1** Relative abundances and comparative analysis of the annotated KEGG functions of gut microbiota in female and male subjects. The KEGG functions with significant differences between groups are presented by Wilcoxon rank-sum test. ^#^P < 0.1, ^*^P < 0.05, ^**^P < 0.01.
